# Towards 90-90-90 Target: Factors Influencing Availability, Access, and Utilization of HIV Services—A Qualitative Study in 19 Ugandan Districts

**DOI:** 10.1155/2018/9619684

**Published:** 2018-03-21

**Authors:** Francis Bajunirwe, Flora Tumwebaze, Denis Akakimpa, Cissy Kityo, Peter Mugyenyi, George Abongomera

**Affiliations:** ^1^Department of Community Health, Mbarara University of Science and Technology, P.O. Box 1410, Mbarara, Uganda; ^2^Joint Clinical Research Center, P.O. Box 10005, Kampala, Uganda

## Abstract

**Background:**

UNAIDS has set a new target 90-90-90 by 2020. To achieve this target, current programs need to address challenges that limit access, availability, and utilization of HIV testing and treatment services. Therefore, the aim of this study was to identify the barriers that influence access, availability, and utilization of HIV services in rural Uganda within the setting of a large donor funded program.

**Methods:**

We conducted key informant interviews with stakeholders at the district level, staff of existing HIV/AIDS projects, and health facilities in 19 districts. Data were also collected from focus group discussions comprised of clients presenting for HIV care and treatment. Data were transcribed and analyzed using content analysis*. Results*. Barriers identified were as follows: (1) drug shortages including antiretroviral drugs at health facilities. Some patients were afraid to start ART because of worrying about shortages; (2) distance and (3) staffing shortages; (4) stigma persistence; (5) lack of social and economic support initiatives that enhance retention in treatment.

**Conclusions:**

In conclusion, our study has identified several factors that influence access, availability, and utilization of HIV services. Programs need to address drug and staff shortages, HIV stigma, and long distances to health facilities to broaden access and utilization in order to realize the UNAIDS target.

## 1. Background

In order to achieve an AIDS-free generation, the UNAIDS has set an ambitious target code named 90-90-90, which aims to ensure that 90% of all people living with HIV will know their status, 90% of all people diagnosed will receive sustained antiretroviral therapy (ART), and 90% of all people receiving ART will have viral suppression, all by 2020 [1]. To achieve this target, countries will need to review the current programs to identify the potential barriers that might hinder the achievement of these goals. In Uganda, a country historically hit hard by the epidemic, progress has been made but more is left to be done to achieve these UNAIDS targets. The adult HIV prevalence is still high at 6.2% in the general population, based on the 2017 national serosurvey [2]. According to the Uganda AIDS commission, only 51% of adults aged from 15 to 49 have taken an HIV test in the past 12 months and know their serostatus [3] and estimated 57% of HIV positive adults are on ART [4] and national population level viral load suppression is estimated at 60% [2] far below the UNAIDS targets.

Current data show numerous socioeconomic and cultural barriers inhibiting access to services and retention in care and these include difficulties in reaching clinics and poverty due to lack of social and financial support [5]. These barriers have been documented in Uganda but mostly within the context of research intensive settings [6, 7]. More studies need to be done in diverse settings so as to obtain results with wider external validity. Particularly, these barriers need to be studied in real life settings and therefore programs that are ongoing provide an ideal framework to study these barriers.

Under these programs, research needs to be done to identify specifically the barriers along the care cascade. These barriers will influence decisions to be tested or not, start with treatment, or stay on treatment. Research has shown that assessing the potential impact of these barriers can tailor improvement in service provision and achievement of treatment goals which include viral load suppression [8, 9], one of the 90-90-90 targets. Data generated can be used to understand challenges faced by HIV positive clients and be used to design culturally and locally appropriate interventions that could be tested. For instance in Mozambique, a qualitative study has shown that mobile ART clinics can reduce barriers to accessing treatment in rural areas [10].

Ongoing projects provide opportunity to study challenges of health programming. The Strengthening Civil Society for Improved HIV/AIDS and Orphans and Vulnerable Children Service Delivery in Uganda (SCIPHA) project is a large community based project based in 19 districts. The project provided opportunity to study challenges of service delivery in a rural setting. Therefore, the aim of this study was to assess the individual, health facility, and community barriers that affect availability, accessibility, and utilization of services in the target districts. We explore factors that influence availability, access, and utilization of HIV services in rural Uganda within the setting of a larger donor funded program in order to provide data that programs can use to improve service delivery in order achieve the 90-90-90 UNAIDS target.

## 2. Methods

### 2.1. Study Setting

The Strengthening Civil Society for Improved HIV/AIDS and Orphans and Vulnerable Children Service Delivery in Uganda (SCIPHA) is a 5-year project which commenced in early 2011 and is being implemented by a consortium comprised of the Joint Clinical Research Centre (JCRC) and Uganda Health Marketing Group (UHMG) in 5 regions of Uganda, namely, West Nile, North, Central, Midwest, and Eastern regions with a total of 19 districts. The districts were all predominantly rural, remote, and underserved, and for northern Uganda many districts here are in a postconflict period after several years of civil strife. The project implements programs through supporting civil society organizations (CSOs) that have grass roots reach. Data were collected in the 19 districts at the inception of the program as part of a larger survey to provide baseline data for program evaluation and insights into the potential challenges in the implementation process. Some of the data from this survey have been published elsewhere [11].

In Uganda, HIV care and treatment services are widely available at many health facilities. HIV testing and antiretroviral therapy are available at facilities as low as subcounty level or health center III. Medical male circumcision is available at county level or health center IV level.

### 2.2. Sampling

We randomly selected one health facility from a list of facilities at county level (health centre IV) in each of the districts. On the data collection days, clients at the HIV clinic were informed about the study and those who showed interest received more information and were asked to consent to participation.

### 2.3. Data Collection

We conducted key informant interviews (KIIs) and focus group discussions (FGDs) in the project districts using pretested tools. Data were collected by a team of research assistants who received training at JCRC. A team of 10 research assistants conducted data collection in the study districts. We needed a large number of RAs because of the diversity of languages in the study districts. The RAs had a basic degree in social sciences or other humanities, had prior experience in collection of qualitative data and for this study, and received training for 5 days that included role plays. The KIIs were conducted in English and FGDs in the local languages of the study districts.

The KII were conducted with key stakeholders at the district level (district health officers, heads of health center IV, and local government) and staff of existing HIV/AIDS projects and health facilities that operate within the targeted districts at district hospitals or health center IVs (county level facility). The study key Informants were purposively chosen for their expert knowledge of the subject being explored. For each interview, at least two researchers participated with one taking the lead on asking relevant questions while the other took the notes; the aim was to minimize the duration of the interview since many of the key informants had very busy schedules and could only afford a limited amount of time for the interviews.

We conducted focus group discussions (FGDs) with clients presenting for HIV care and treatment services at the health facilities. Data were collected till saturation was achieved. Typical questions asked in the KIs and FGDs were “Are services for HIV testing readily available, are antiretroviral drugs continuously available, Do you have any worries that shortages for your HIV medicines will happen while you are on treatment, Are there any services for HIV care that should be provided that are not available and are you concerned about privacy when you come to the clinic for services?” Data were collected between September and December 2011, audiotaped, and transcribed verbatim in preparation for analysis. Analysis was done manually.

#### 2.3.1. Qualitative Description of Study Variables

*Availability* refers to the physical access or reach of HIV/AIDS services that meet a minimum standard.* Access* was generally referred to as the presence of physical or economic barriers that people might face in the use of health services. Physical barriers were those related to the general supply and availability of health services and distance from health facilities. Economic barriers were those related to the cost of seeking and obtaining healthcare, in relation to a patient's or household's income.* Utilization *refers to process of patients visiting health facilities or service delivery point, to use available HIV/AIDS services.

### 2.4. Data Analysis

Textual data were then explored using inductive content analysis. Data were read and reread by three experienced analysts (FB, FT, and AG) who were also involved in the data collection. The data were reread in order to identify the emerging themes and categories from the transcripts [12]. All data relevant to each category (availability, access, and utilization) were identified and examined using the process of constant comparison, in which each item was compared with the rest of the data to verify its category. For those sections of the data that included multiple themes, these were coded using several categories using process indexing [13]. This process allows the analysis of data items to fit into several categories. Typical quotes were also collected and included in the manuscript in order to emphasize the response given without losing the original context of the meaning.

### 2.5. Ethical Considerations

This study involved interviewing human subjects and, therefore, ethical approval to undertake the study was obtained from the Uganda National Council of Science and Technology (UNCST). We also sought permission from the Chief Administrative Officers of the study districts. Before each interview, the objectives of the study were explained to the respondents to enable them provide verbal informed consent. Informed consent was also obtained to make audio recording of the interviews. Potential respondents were made aware of their rights to refuse the interview, or to refuse to answer specific survey questions, without any implications on their access to care.

## 3. Results

### 3.1. Demographics

We conducted 32 KI with health officials in the study districts and none of those approached to participate declined. Eighteen or 56% of them were male, the majority had a university degree with more than 5 years of experience in HIV related work. The characteristics of the informants are shown in Table 1.

A total of 38 FGDs were conducted at 19 sites. An FGD had on average from 8 to 12 clients and lasted about one hour.

### 3.2. Overview of Results

Three themes formed the basis of the analysis and results are organized under these three subheadings: availability, access, and utilization of HIV/AIDS services.  Table 2 shows a section of the questions that were asked, codes, and the emergent themes under which they belong.

#### 3.2.1. Barriers That Affect the Availability of HIV/AIDS Services in the SCIPHA Districts

In the KI interviews, participants were asked about the availability of HIV testing and treatment services including services such as test kits and HIV medicines. The majority of respondents indicated that the major barrier was a general problem of drug shortages at the health facilities. Most notably, the shortages involved antiretroviral drugs and Cotrimoxazole. Additionally, there were no means of communication to inform clients whether the HIV/AIDS drugs are in stock or not. In the FGDs, participants also alluded to the drug shortages. They mentioned that clients often resort to buying Cotrimoxazole from pharmacies and clinics as a coping mechanism. Some participants reported buying drugs from the markets on market days because they were much cheaper there. Market days are weekly gatherings, common in rural and periurban Uganda, where traders and vendors converge in designated open spaces to display and sell their merchandise ranging from foodstuffs to clothing and electronics, attracting large crowds. This is illustrated by the typical quote below:

*We have no drugs in our hospital- the little medicine that exists is also sold to us by the medical people*. (FGD participant, northern Uganda)

*We walk long distances to access the health centres in the district, so finding when there is no medicine is discouraging*. (FGD participant, Eastern Uganda)

FGD participants mentioned that because of the common occurrence of drug shortages at health facilities, some health workers hesitate to initiate patients on antiretroviral therapy. They mentioned that supply was often inconsistent and that health workers were aware that, for ARVs, one needs to take the drugs for life. When asked whether they would start ART with an uncertain supply of medicines, a focus group discussion member in a district in Eastern Uganda emphasized their fears:

If you start medication and then fail to keep taking it, it shortens your lifespan. So it is better not to start at all when you are not sure of supply.

The FGD participants also indicated that the shortage of health workers limited the availability of services as mentioned by a female FGD participant:

Sometimes there is no doctor in the hospital, usually it is the midwife only and even then she has to be begged to help.

There was an imbalance between prevention, treatment, and other support services. Key informant interviews with HIV/AIDS service providers showed that more of the service providers offer HIV prevention services compared to care and treatment and and social support as one KI stated in the interview.

*I think we have done a good job in providing Information, education and communication. Condoms are widely available but we have not given the same attention to treatment and the numbers are growing*. (KII Eastern Uganda)

The KI reported that the availability of social support services was generally low in the SCHIPA project districts, yet these services might help retain their clients in the treatment program. Very few service providers were reported to offer services such as income generating activities (IGAs) for people living with HIV/AIDS and spiritual and social support. They also mentioned that for the youth, there was need to have youth friendly treatment and information centers where young people can visit more freely.

The key informant interviews showed that there were currently very few interventions aimed at prevention of HIV transmission among discordant couples. The KII indicated that clients already enrolled in treatment demand services that provide care for the whole family. The respondents had the belief that a holistic family approach might draw more clients into care and treatment. When asked about services that they would wish to provide to engage the whole family, one KI mentioned:

For example, a client will tell you that my wife is HIV negative and I don't want to infect her, but I also get tired of condoms. What can I do?

#### 3.2.2. Barriers That Affect Access to HIV/AIDS Services in the Study Districts

The majority of the HIV clients reported that services at the clinics were offered at inconvenient hours and days to the clients. Many of the HIV/AIDS care centers in the project districts provide HIV clinics once a week. They expressed a desire to have alternative days. Laboratory equipment to measure CD4 count is only available in selected districts. In some of the districts where the equipment is available, other challenges emerge as illustrated in one of the KI interviews:

*For some districts like (Y) When we ask, CD4 count services are only provided every 4 months. For other districts, there is rarely power to run the CD4 machines and yet there are no generators at the facilities*. (KI interview)

In some situations, the service is available but other logistical issues make the service inaccessible. For instance, when one FGD was asked what would prevent a client from having a CD4 count measurement given that the machine was available and functional, they mentioned challenges as illustrated by the quote below:

*If one goes to (district X) for the CD4 count - it is very challenging. The blood sample is collected in the wee hours of the morning at 4:00am. So one has to travel the day before and spend a night in the hospital if their blood sample is going to be taken. That whole process is a pain. But what do you do, we do not have any alternative.* (FGD participant in District X, Eastern Uganda)

Another FGD participant in District D had the following to say.

The health centre lacks machines for CD4 count and therefore our blood samples have to be taken to Z (over 150kms away). Sometimes the results for the CD4 count get lost in the process.

In District C, a similar complaint was reported as indicated in the quote below.

*For the CD4 count machine, every patient pays 10,000/= and those who can't afford, do without because as a district, we don't have a CD4 count machine *(KII, District C, western Uganda)

The majority of the services are facility based and there are few opportunities for outreach services in the community. FGD participants generally expressed the need to be followed up in their homes and monitored. They mentioned that bringing the medicines closer would reduce the transport costs and also allow them to tend to their gardens and businesses without interruption. However, some mentioned that sometimes illness prevents one from being able to travel as illustrated below:

*Sometimes you are so weak you cannot make the journey to the health facility and it would be nice if health workers can bring medicines to your home. When some patients become too sick to travel, they resort to going to traditional health workers for assistance*. (FGD participant, District D)

Within the SCIPHA project districts, some Civil Society Organizations (CSOs) covered only a small proportion of the communities, such as one or two parishes in an entire district. One KI mentioned the following:

*This leaves many parishes and sub-counties with no grass roots service. The absence of these CSOs in many areas is compounded by the poor road networks in addition to the long distances of travel,…….. making access to the HIV/AIDS service centers difficult, especially in the hilly regions*. (KII District D)

In most of the project districts, the roads were reported to be inaccessible and muddy especially during the rainy season.

#### 3.2.3. Barriers That Affect the Utilization of HIV/AIDS Services in the Target Areas

Stigma was mentioned as a major barrier in this study. There still exists a lot of stigma in the communities regarding HIV/AID and this was mentioned in all the study districts. As a result, some clients fear to be tested or go for treatment at health facilities. An FGD participant in District X when asked why some persons do not test reported the following:

HIV is a feared disease in our community. People do not want to test for HIV, and those who have tested and found themselves positive have refused to start treatment…. I think the main reason for not wanting to test for HIV or to start treatment is that the people do not want others to know that they are HIV positive.

In the FGDs, respondents reported that women more often test for HIV but do not disclose their status to their husbands because of fear of domestic violence and separation. The respondents mentioned that it was common to have a couple unaware of each other's HIV status. For the women, it seemed to have more serious consequences because they will not seek treatment for fear of having their HIV status disclosed.

Distance was mentioned as another major barrier to utilization of services by a large majority of respondents. FGD participants repeatedly mentioned poverty as a factor making the availability of these services even more difficult. They mentioned that patients need money to pay for transportation to the health facilities, buy drugs when there is shortage, and also buy food necessary to maintain a well-balanced meal. This was illustrated in the quote below from an FGD participant in District Q, Eastern Uganda:

*I live far and I have to save money for transport over the whole month in order to get here………..And some people on ARVs can barely afford the kind of nutrition these drugs need because these drugs are so strong. They (patients) sometimes take drugs on empty stomachs and this makes things worse*. (FGD participant in District Q, Eastern Uganda)

The majority of heads of the health centers indicated that there was a severe staff shortage. They mentioned that only a few health workers were available to serve the many HIV/AIDS clients on the clinic days and this was reported to be the cause of the long waiting times. They mentioned that health workers get overwhelmed by the patient numbers and get very fatigued and stressed and as a result might become rude to the patients. When asked about personal experiences or such encounters with health workers or facilities where they were treated badly, one FGD participant cited an experience:

The attitude of the doctors when we try to raise complaints about the lack of staff or delays is very bad. One health worker told me, ‘atalina maanyi tagwa ddalu', implying that one without money shouldn't fall sick…….

FGD participants reported that improper treatment of patients by the healthcare providers was not uncommon. Some respondents mentioned that this might prevent some clients from seeking care at the health facilities whenever they need it. Participant also complained that there were not enough counselors at most health facilities which also contribute to long waiting times at the service delivery points.

The problem of long waiting time is aggravated by the inability to get food or snacks to eat when hungry, on the clinic days. This has forced some clients not to seek services as illustrated by one respondent:

*You go to the hospital at 8am and you leave at 6pm…. and there is nothing to eat….so someone chooses not to come back.* (FGD participant, District J)

Clients were also concerned that, in some facilities, health workers did not give them adequate time. One FGD participant in District J had the following to say.

*Service providers report at 10am and by 2pm they have left the health centres. If you are not there within that time, you miss and then have to come back*. (FGD participant, District J)

*At the Health Centre, there are few health workers and many patients. A whole day like today, the health workers work on only 30 patients and the rest go home unattended.*

There were some concerns about privacy at some of the sites, and some clients did not feel that they had sufficient privacy during their contact with health workers. The counseling room for persons testing for HIV was shared with those on ART due to insufficient space at the health facility.

## 4. Discussion

Our study has identified several factors that influence availability, access, and utilization of HIV related services. For availability, the factors include drug shortages at the health facilities, lack of material and psycho-social support services and specialized services such as pre-exposure prophylaxis. Among the responses, drug shortages emerged as a major factor. Drug shortage prompts patients to raise concerns even about starting ART, with a worry that drugs might eventually run out. Couples in HIV discordant relationships have specific needs that programs are currently not addressing. For access, HIV clinics and laboratory testing are accessible only once a week at many sites and for some districts at very few sites. There was absence of grass roots level service providers. For utilization, stigma causes fear to test and disclose HIV serostatus. Distance to health facilities remains a barrier: shortage of health workers and long waiting hours and inadequate privacy at the health facilities prompt some patients not to use the services.

Our study showed that distance is a major barrier influencing access to services. The majority of participants in the FGDs agreed that this was one of their biggest challenge. In some situations, the medications and other services are available, but the terrain makes it very difficult to access treatments. We heard in the FGD that some patients had to travel a day before in order to arrive in time for a blood collection to have their CD4 counts estimated. Long distance is still a major structural barrier limiting access to treatment. Our findings are similar to those from another Ugandan study [7] and another in Tanzania [5] which showed distance as a major barrier for access to care and retention. In fact distance has been reported to be a barrier in several other studies [14, 15]. A review of studies on barriers to ART care showed that distance and transport costs were the most commonly cited barrier [16]. Participants generally expect that health facilities should be within a manageable distance to ensure regular attendance at the clinics. As distance was noted to be a barrier, programs will require interventions such as mobile pharmacies that have been shown to reduce distances travelled [17].

Drug shortages are a common occurrence in many health facilities in resource limited settings [18] and not just for antiretroviral therapy but for other medications as well [19, 20]. Drug shortages or unavailability has been associated with increased risk of loss to follow up and mortality at HIV clinics in West Africa [21]. In our study, unavailability of antiretroviral drugs and Cotrimoxazole were reported in almost all the project districts. It is against this backdrop that some patients in our study expressed fear at initiating treatment. They carried concerns that medications may run out some day and their prognosis would be worse than if they had not started treatment at all. These findings are not unique to our study. In Zambia, a study to determine barriers of ART initiation found concerns about long-term availability of these medicines but also found other factors such as belief that ART was a “strong” medicine and hence one needed to have a “strong body” to take it and other reasons such as lack of self-efficacy and a desire for normalcy, which hindered initiation of treatment [22]. We were not able to elicit all these factors, but because of the similarity of the settings, these need to be considered in programs in other resource limited settings.

The SCIPHA project supports CSOs, which are community based entities, and hence it provides service at the grass roots. However, our data have shown that there are a limited number of service providers who offer HIV/AIDS training and support with school based interventions like life skills training for youth, especially targeting adolescents, who are a high risk group. The youth are also likely to be in need of social support services including income generating activities which were generally not available in the study districts.

There were a limited number of health workers trained in HIV/AIDS care at several rural sites. Some of the service delivery points that were visited had insufficient numbers of trained health workers to serve the many HIV/AIDS clients on the clinic days and this was reported to be the cause of the long waiting times. There was also limited availability of services which target the most at risk populations (MARPs) or are friendly to the specific MARPs. Very few health facilities were reported to provide safe male circumcision (SMC) as part of the package for HIV prevention services. The demand for these services is high; hence programs need to scale up training of cadre that can safely conduct SMC. Lower cadre staff like nurses and counselors may be trained and supported to offer these services. This is critical as universal test and treat strategy have been fronted as keys in achieving the 90-90-90 goals and ending AIDS [23].

An important new finding in this study was that there is a demand for Preexposure prophylaxis (PreP) among some clients in discordant relationships. There were very few service providers that reported offering antiretroviral therapy for Postexposure prophylaxis (PEP) and even none for PreP. The World Health Organization has issued guidelines on PreP for implementation but many countries including Uganda have not made bold steps in the implementation. Programs should quickly design measures to incorporate and integrate PreP into current programs.

Patients lacked knowledge and carried several misconceptions related to HIV disease and treatment. Patients in our study were still dealing with the stigma associated with HIV disease. In a large systematic review of barriers to HIV care, stigma was cited as the second most common barrier [16]. Antiretroviral therapy helps to restore physical functioning of HIV patients and therefore helps to reduce stigma, but studies show that many patients continue to conceal their status because of fear of rejection, gossip, and anticipated stigma [24–26] especially among women and persons with disabilities [27]. Fear of stigma persists especially among those who initiate new sexual relationships or new jobs and those who develop side effects [28]. Interventions against stigma need to be designed especially after additional data show that stigma is more concentrated among persons in the lowest socioeconomic categories [29]. These interventions should also put into consideration other factors that influence stigma such as spiritual beliefs [30] and special cases such as women in antenatal care [31].

Some respondents mentioned that fear to start antiretroviral therapy may be because of lack of food and the belief that the drugs are strong, might overpower their bodies, and therefore require special nutrition. Families affected by HIV suffer significant food insecurity. Several intervention trials on food security among HIV patients are currently underway and will provide valuable data on the impact [32, 33]. Early results from a pilot study in Honduras have shown that household food support may improve food security and nutrition education alone was associated with weight gain [34]. The Shamba Maisha project in Kenya, a randomized controlled trial of an intervention involving agriculture and financial assistance, showed significant increases in the CD4 count, viral load suppression, and food security in the intervention arm compared to the controls [35].

The increasing numbers of patients at HIV clinics have created overcrowding and long waiting times. The resulting delay at the clinic is a deterrent and may present a potential barrier to initiation and continuation of ART. There is a major shortage of healthcare workers in Uganda with one doctor for a population of over 24,000 residents [36]. The human resources for health crisis in HIV care may be mitigated by task shifting. Task shifting has been shown to be cost effective without compromising patient outcomes [37–40]. Data from other countries in Africa show that stakeholders agree that task shifting aids in increasing access to ART, decreases workload, and lowers patient wait times [41]. Studies should be done to evaluate different forms of task shifting involving variation in the nature of task and/or cadre of staffing involved. Several countries including Uganda have no policy on task shifting and there are concerns that tasks may be shifted to incompetent and already overworked staff, hence defeating the purpose [42, 43].

We did not find any region specific challenges. Respondents from northern, western, and central Uganda where the study was conducted shared similar experiences. This implies that patients across the country in rural Uganda, regardless of location, face the same challenges. Similar studies to assess barriers to achieving the UNAIDS 90-90-90 targets have been done elsewhere and also agree with our findings. For instance in the Asia Pacific region, the barriers identified were similar to those in our study and were classified as political, demographic, socioeconomic, and health system factors [44].

An important strength of our study is that we were able to collect data within diverse regions across the country; hence our data is rich in scope and context. One major weakness is that we did not collect demographic characteristics and other important features of the FGD participants. Lastly, although these data were collected over 6 years, the challenges and gaps presented here still persist to date and can only be exacerbated by demands of the new test and treat policy.

## 5. Conclusion

In conclusion, our study has identified several factors that influence access, availability, and utilization of HIV services. They are mostly structural barriers that include transport and distance to health facilities, health system factors like human resources for health, and quality of health services being provided. Our study has made recommendations, such as provision of on-demand services, task shifting, and financial support, aimed at improving access, availability. Programs also need to deal with stigma and other barriers that limit utilization of these services in order to achieve the UNAIDS 90-90-90 target. Community based controlled studies need to be done to test these proposed interventions.

## Figures and Tables

**Table 1:** Characteristics of Key informants in the Qualitative study in the 19 SCIPHA districts in Uganda.

Characteristic	*n* = 32 (100%)
*Gender*	
Male	18 (56.3)
Female	14 (43.7)
*Age category in years*	
Less than 30	6 (18.7)
30 to 40	21 (65.6)
Over 40	5 (15.7)
*Employment Category*	
District Health officer	7 (21.8)
Health center in charge	12 (37.5)
HIV program	8 (25.0)
Local government	5 (15.7)
*Years of experience in HIV related work*	
Less than 2	3 (9.4)
2 to 5	12 (37.5)
Over 5 years	17 (53.1)
*Educational level*	
University degree	25 (78.1)
Diploma	5 (15.6)
Other	2 (6.3)

**Table 2:** Section of the questions, meaning unit, condensed meaning unit, codes, and themes from qualitative survey among respondents in 19 districts in Uganda.

Questions	Meaning unit	Condensed meaning unit	Codes	Category/Theme
Are services for HIV testing, treatment, monitoring available when one needs them	If you start medication and then fail to keep taking because of drug shortages, it shortens your lifespan. So it is better not to start at all	(i) If you start medicines, you can't stop (ii) Drug shortages occur (iii) You can die if you stop medicines	(i) Drug shortages (ii) Continuous medication (iii) Fear to start ART	Availability of HIV services

Would every patient be able to get the same service at the HIV clinic?	For the CD4 count machine, every patient pays 10,000/= and those who can't afford, do without because as a district, we don't have a CD4 count machine	(i) Patients pay for CD4 count testing (ii) District lacks testing equipment	(i) High cost of testing (ii) Lack of equipment (iii) Equipment break down	Accessibility of HIV services

Why do some clients not take advantage of the available services such as HIV testing?	I think the main reason for not wanting to test for HIV or to start treatment is that the people do not want others to know that they are HIV positive	(i) Some clients do not want to test for HIV (ii) Fear to start treatment (iii) Clients do not want their HIV serostatus known	(i) Stigma (ii) Fear to disclose serostatus	Utilization of HIV services
